# Enhancement of *Calibrachoa* growth, secondary metabolites and bioactivity using seaweed extracts

**DOI:** 10.1186/s12906-016-1332-5

**Published:** 2016-09-02

**Authors:** Hosam O. Elansary, Jeff Norrie, Hayssam M. Ali, Mohamed Z. M. Salem, Eman A. Mahmoud, Kowiyou Yessoufou

**Affiliations:** 1 Biodiversity Institute of Ontario, University of Guelph, Guelph, Ontario N1G 2W1 Canada; 2 Department of Floriculture, Ornamental Horticulture and Garden Design, Faculty of Agriculture (El-Shatby), Alexandria University, Alexandria, Egypt; 3 Acadian Seaplants Limited, 30 Brown Avenue, Dartmouth, Nova Scotia Canada; 4 Botany and Microbiology Department, College of Science, King Saud University, P.O. Box 2455, Riyadh, 11451 Saudi Arabia; 5 Timber Trees Research Department, Sabahia Horticulture Research Station, Agriculture Research Center, Horticulture Research Institute, Alexandria, Egypt; 6 Forestry and Wood Technology Department, Faculty of Agriculture (EL-Shatby), Alexandria University, Alexandria, Egypt; 7 Department of Food Technology, Damietta University, Damietta, Egypt; 8 Department of Environmental Sciences, University of South Africa, Florida campus, Florida, 1710 South Africa

**Keywords:** *Calibrachoa*, Seaweed extract, Antifungal, Antibacterial, Antioxidants

## Abstract

**Background:**

*Calibrachoa x hybrida* (Solanaceae) cultivars are widely used in North and South America as ornamental plants. Their potential as a source of antimicrobial compounds might be enhanced by seaweed extract (SWE) applications.

**Methods:**

SWE of *Ascophyllum nodosum* were applied at 5 and 7 ml/L as a soil drench or foliar spray on *Calibrachoa* cultivars of Superbells® ‘Dreamsicle’ (CHSD) and Superbells® ‘Frost Fireʼ (CHSF). The total phenolics, tannins and antioxidants composition as well as specific flavonols in leaf extracts were determined. Further, the chemical composition of SWE was assessed.

**Results:**

The drench and foliar SWE treatments significantly enhanced *Calibrachoa* cultivars leaf number and area, dry weight, plant height, antioxidant capacity as well as phenolic, flavonols and tannin content. The increased growth and composition of phenols, flavonols and tannins was attributed to the stimulatory effects of SWE mineral composition. The antifungal activity of *Calibrachoa* cultivars was significantly enhanced following SWE treatments and the minimum inhibitory concentration (MIC) and minimum fungicidal concentration (MFC) were in the range of 0.07–0.31 mg/ml and from 0.16 to 0.56 mg/ml, respectively. Moreover, antibacterial activity was significantly increased and the MIC and minimum bactericidal concentration (MBC) measurements were in the range of 0.06–0.23 mg/ml and from 0.10 to 0.44 mg/ml, respectively. The most sensitive fungus to SWE treatments was *C. albicans* and the most sensitive bacterium was *E. cloacae*.

**Conclusion:**

The results suggest that enhanced antifungal and antibacterial activities might be attributed to significant increases of phenolic, flavonols and tannin contents, which ultimately enhance the potential of *Calibrachoa* as a natural source of alternative antibiotics.

## Background

Phytopathogens such as fungi and bacteria can cause serious diseases to humans and lead to large losses in agricultural crops production [1–3]. Recent studies show that natural plant products are safer alternatives to antibiotics and commercial pesticides that are commonly used to control these pests [4, 5]. Enhancing the plant’s natural photochemistry might therefore serve as a useful technique to increase the production of natural defensive compounds and safely control diseases. In this respect, seaweed extracts (SWE) which are obtained from algal species growing along coastal regions around the world, might be used as natural plant biostimulants [6]. These extracts are usually associated with enhanced plant growth following foliar or drench application [7, 8]. However, horticultural crops exhibit a range of diverse responses following different application methods and doses of SWE [7, 9]. Few reports on vegetable crops indicate that SWE treatment may affect the crop nutritional composition by means of increasing phenolics, flavonoids, and antioxidant capacity [10, 11]. Antioxidant activities are commonly associated with phenolic and flavonoid content in medicinal shrubs [12] and in plants in general [13, 14]. Furthermore, little attention has been given to ornamental medicinal plants [15, 16].

In addition, the antifungal and antibacterial activities of SWEs are widely demonstrated and these activities are mostly linked to the presence of thermo-stable lipophilic compounds [17–20]. For example, some SWEs have bactericidal effects [18] associated with the presence of terpenes and phenolic compounds [17, 19]. Other reports indicate that seaweeds might stimulate the production of plant defense elicitors (e.g. oligosaccharides) against pathogens [21]. SWE foliar spray may also stimulate microbes antagonistic to *Pythium ultimum* [22]. Further, SWE foliar sprays on turfgrasses may enhance resistance to dollar spot [6, 17]. Many investigations also indicate that plant extracts have useful value in controlling microorganisms [4, 5] which may allow for a reduction in the application of fungicides and bactericides, and consequently minimize their potential side effects on human health and the environment. Nevertheless, how to use SWE as a biostimulant of natural phytochemistry production to enhance the medicinal values of plants is a poorly investigated question. To our knowledge, the effects of SWE on plant extract activities against microorganisms had not been investigated in vitro.

Solanaceae is a large plant family that contains many horticultural crops that are of economic and medicinal importance such as the *Petunia* and the recently delimited genus *Calibrachoa* [23–25]. *Calibrachoa* cultivars are widely grown by seed in North and South America from hybrid cultivars [26]. Some *Calibrachoa* cultivars vary in their content of phenolic, flavonoid and tannin compounds and their leaf extracts may exhibit antifungal and antibacterial activities against a wide spectrum of pathogenic microorganisms [27], further, close genera to *Calibrachoa* such as *Petunia* had shown obvious flavonoid composition [28] and antimicrobial activities [29], however little is known about Solanaceae members such as *Calibrachoa* responses to SWE from the point of chemical composition.

In the current study, we investigate the effects of SWE of *Ascophyllum nodosum* on enhancing *Calibrachoa* plants medicinal value by way of increasing the phenolic, flavonoid and tannin contents as well as their antioxidant capacity. Further, we examined the use of SWE technologies to enhance antifungal and antibacterial activities of plants against a selected spectrum of microorganisms. This research may represent an important methodology towards enhancing the quantity and quality of natural products obtained from promising sources of natural products such as *Calibrachoa*; it could also lead to reducing the use of antibiotics and commercial reagents in agricultural crop disease and pest control programs, thereby minimizing their impact on human health and our environment.

## Methods

### Chemicals and cell cultures

Analytical/HPLC grade chemicals were obtained from Sigma Aldrich, Canada. Fungi and bacteria were obtained from Henry farms Laboratories, Guelph, Canada.

### Plant material and SWE treatments

Uniform 2 weeks old plants of *Calibrachoa* x *hybrida* of ʻSuperbells® Dreamsicleʼ (CHSD) and ʻSuperbells® Frost Fireʼ (CHSF) were obtained from local nurseries, identified by Dr. Hosam Elansary, University of Guelph, and vouchered at the Biodiversity Institute of Ontario, University of Guelph, Guelph, Canada. The plants were subjected to a weekly treatment of the commercially available liquid extract of the marine plant *Ascophyllum nodosum* (Stella Maris^TM^, Acadian Seaplants, Canada) at 5 and 7 ml/L per plant as a soil drench or foliar spray. Untreated plants were considered as controls. SWE treatments continued for 8 weeks before sampling plant leaves. *Ascophyllum nodosum* was harvested from the intertidal zone along the North Atlantic coastline of Canada and then hydrolyzed under atmospheric pressure using KOH. The liquid extract was evaporated under vacuum to a concentration of 29 % solids.

### Plant material, growing conditions and morphological parameters

The plants were transplanted into a simulated green roof pot system in commercial 4-in. pots measuring 13 cm in height and equipped with a filter (fabricated non-woven geotextile of polypropylene) and 4 cm drainage layers of gravel (0.5 ml). Pots were arranged during April 2015 in a controlled greenhouse environment located in Guelph, Ontario, Canada (43° 30′ 18.24″ N 80° 22′ 15.86″ W). The substrate used for the green roof system was black peat and perlite (3:1 w/w) and was supplemented with Osmocote Plus® (14:13:13 N, P, K + microelements) (2 g/l media). The experiment was conducted under the following conditions: Temperatures ranged between 19.6 °C and 27.4 °C; relative humidity ranged between 55 and 62 %; photosynthetically active radiation was maintained at 1000 μmol/m^2^/s at 10.00 am, and plants were maintained at 12 h light conditions for 1 week before starting treatments. A daily watering (25–40 ml) was applied to allow for 100 % evapotranspiration rate (ET). ET was determined for 5 plants per cultivar by watering with enough water and leaving them to dry for 1 h, then weighing each representative, reweighing every 24 h, and the daily changes in weight represent the daily ET. The volumetric water content was calculated for 5 pots before and during the experiment by determining the weight before and after irrigation by allowing free draining for 1 h. The difference between the fresh weight and oven-dry weight (at 70 °C until constant weight at the end of the experiment) was calculated to provide the volumetric water content. Plants were grouped into three blocks/repetitions (*n* = 3) containing 4 replicates per treatment in the experiment and totaling 120 plants per cultivar distributed on three plots. After 8 weeks of treatments, the plants were harvested and plant height and leaf number were calculated. A WinDIAS Leaf Area Measurement System (Delta-T Devices Ltd., Cambridge, UK) was used to calculate the leaf area. Total dry weight was determined by drying cleaned plants in an oven at 70 °C to reach constant weight.

### Preparation of leaf extracts

Leaf samples of *Calibrachoa* cultivars were collected in June 2015 and leaves’ extracts were obtained using methanol as per Pérez-Tortosa et al. [30] with some modifications [31]. Dried leaves (0.25 g) were ground then dissolved in 3 ml methanol (99 %). The solution was shaken on a magnetic agitator at minimal speed under darkness (to maintain the activity of bioactive compounds), for 24 h at room temperature. The solution was centrifuged for 5 min (4°C) at 10000 RPM (7000 × *g*) and the supernatant (~2.6 ml) was stored in sealed vials at −20 °C. The preparation was evaporated in a rotary evaporator to produce a semisolid extract that was kept for further analyses.

### Antioxidant capacity

The 2,2′-diphenypicrylhydrazyl (DPPH) method was employed to determine the free radical scavenging activity of the samples [32]. The absorbance was measured at 517 nm and experiments were repeated twice in triplicate. The *β*-carotene-linoleic acid assay was conducted using the method described by Tepe et al. [32] with modifications [27]. The mixture of *β*-carotene-linoleic acid was prepared by dissolving 0.5 mg *β*-carotene in 1 ml of chloroform, 25 μl linoleic acid and 200 mg Tween 40. Chloroform was removed by vacuum evaporation then 100 ml of distilled water saturated with oxygen were added and shaken vigorously. 2.5 mL of the mixture was mixed with 350 μl of the liquid extracts, incubated for 48 h at room temperature and the absorbance was measured at 470 nm. The BHT was used as positive control and a blank was prepared in the same manner and the antioxidant capacities of each sample were compared to the BHT and the blank. Antioxidant activity was expressed as the concentration of the sample required to inhibit 50 % of DPPH or β-carotene-linoleic acid (IC50).

### *Calibrachoa* total phenolic, tannin and major flavonols contents quantification

The Folin-Ciocalteau colorimetric method using gallic acid as the reference was employed to determine the phenolic contents of leaf extracts with results expressed as gallic acid equivalents (mg GAE/g ext.) [33, 34]. Flavonoids were determined in plant leaves by extracting 100 mg ground leaf tissues in 2.1 ml methanol:acetic acid:water (70:4:29, v/v) for 72 h at 4 °C, then the supernatant was removed and the pellet was re-extractred in 2 ml methanol:acetic acid:water (90:1:9, v/v) for 24 h at 4 °C. The combined supernatants were dried (under vacuum) and completed to 0.5 ml with a mixture of methanol:acetic acid:water (80:2:17 v/v) [35]. The samples were subjected to HPLC analysis of flavonoids. The HPLC analysis was performed using liquid chromatographic system equipped with a Waters Alliance 2695 separations module (Waters, Milford, MA, USA). A LiChrosphera RP-18 reversed-phase column (119 mm 4 mm) supplied by Merck (Quebec, Canada) was employed. A gradient solvent system of A [HOAc:CH3CN:H3PO4:H2O (20:24:1.5:54.5)] and B [1.5 % H3PO4] was used starting with 80 % A, decreasing to 30 % A at 30 min, 15 % A at 34 min and 0 % A at 40 min. The flavonoids were determined as quercetin-3-O-rutinoside equivalents by integrating areas and the absorbance was monitored at 352 nm. Tannins were determined following the gravimetric method [36] with modifications [37]. All experiments were repeated twice in triplicate.

### Chemical composition of SWE

Inductively Coupled Plasma Spectroscopic Analysis (ICPSA) was used for SWE to determine the mineral compositions in Optima 4300DV (Perkin-Elmer, USA). The nitrogen content (N) was determined using AOAC method No. 990.03 in the LECO FP-528 analyzer. Phosphorous pentoxide (P_2_O_5_) was determined using the ammonium citrate in AOAC method No. 960.08 by ICP-OES. Potassium oxide (K_2_O) was determined using the ammonium oxalate in AOAC method No. 960.08 by ICP-OES. Heavy metals quantification followed the AOAC method No. 6020A using the Atomic Absorption -Hydride Generation. Experiments were repeated twice in triplicates.

### Antifungal activities

Four fungi were used: *Aspergillus flavus* (ATCC (American type culture collection) 9643), *Candida albicans* (ATCC 26555), *Penicillium funiculosum* (ATCC 56755), and *Penicillium ochrochloron* (ATCC 48663). Cultures were kept on malt agar at 4 °C then sub-cultured monthly. Spore suspension concentration of (1.0 × 10^5^) was maintained and the minimum inhibitory (MIC) and minimum fungicidal (MFC) concentration assays were performed using the microdilution method [38]. Leaf extracts were diluted in 5 % DMSO (1 mg/ml and 10 mg/ml), then added (2 μl) to microplates containing broth Malt medium with inoculum and incubated for 72 h at 28 °C on a rotary shaker. MIC was determined by serial sub-cultivations of 2 μl of leaf extract and isolated compounds dissolved in a medium. The sub-cultivations were incubated for 72 h in microtiter plates that contain 100 μl broth and inoculum, then incubated for 72 h at 28 °C. MIC was defined as the lowest concentration that inhibits fungi growth at the bionocular microscope level. To determine the MFC a serial dilutions of the inoculum was prepared in 96-well microtiter plates and the MFC was defined as the lowest concentration with no visible growth indicating 99.5 % killing of the original inoculum. Triplicate samples were used in all experiments and each experiment was repeated twice. The fungicides fluconazole (FLZ) and ketoconazole (KLZ) were used as positive controls (1–3500 μg/ml).

### Antibacterial activities

Experiments were performed using Gram + Bacillus *cereus* (clinical isolate), *Staphylococcus aureus* (ATCC 6538) and *Micrococcus flavus* (ATCC 10240) and Gram-bacteria *Enterobacter cloacae* (ATCC 35030). The microdilution method [38] was used to determine the minimum inhibitory (MIC) and minimum bactericidal (MBC) concentrations. Bacterial suspensions were adjusted with sterile saline to a concentration of 1.0 × 10^5^ CFU/ml and stored at 4 °C. Inocula were screened for contamination by culturing on a solid medium. Leaf extract were added (1 and 10 mg/ml) to 100 μl Triptic Soy broth (TSB) with a bacteria inoculum (1.0 × 10^4^ CFU/well), reaching the desired concentration in a microtitre plate to measure the MICs and MBCs. The mixtures in microplates were incubated for 24 h at 37 °C in a rotary shaker. After incubation of the microplates, the lowest concentration that completely inhibited bacterial growth (at the binocular microscope) was defined as the MIC. To determine the MBC, a serial sub-cultivations of 2 μL into microtitre plates containing 100 μL of TSB for each well and incubated for 24 h. The MBC was defined as the lowest concentration indicating killing of 99.5 % of the original inoculum. To determine the optical density a microplate manager was used at 655 nm and experiments were in triplicates and repeated three times. The results was compared to positive controls (streptomycin and ampicillin, 1 mg/ml), and negative control (5 % DMSO).

### Statistical analysis

The results were expressed as means ± standard deviation (SD). Further, the data was subjected to the Least significant differences (LSD) one-way analysis of variance (ANOVA) implemented in SPSS (PASW Ver. 21) at a level of significance of *P* ≤ 0.05.

## Results

### Growth, antioxidants, total phenolic, flavonols and tannin contents in *Calibrachoa* and chemical composition of SWE

Foliar and drench SWE treatments significantly enhanced growth parameters (leaf number and area, dry weight and plant height) compared to control plants (Table 1). Treated plants showed higher antioxidant capacity in both *Calibrachoa* cultivars compared to control plants as determined from both DPPH and linoleic acid assays (Table 2). In the DPPH assay, the IC50 ranged from 27.3 to 33 ug/ml and from 29.4 to 37.9 ug/ml in CHSD and CHSF, respectively. The drench application of SWE showed higher antioxidant activity than the foliar ones; also the 7 ml doses of SWE had higher antioxidant activity in leaf extracts than the 5 ml dose. The linoleic acid assay followed a parallel pattern to that found in the DPPH assay. The phenolic content ranged from 4.6 to 5.2 mg GAE/g ext. and from 4.4 to 5 mg GAE/g ext. in CHSD and CHSF, respectively. The total phenolic content significantly increased when increasing SWE dose as well as when using the drench application. However, the 5 and 7 ml drench doses of SWE did not show significant differences in the phenolic content in the CHSF cultivar only.

**Table 1:** *Calibrachoa* cultivars leaf number (leaf/plant), leaf area (cm2/plant), plant dry weight (g/plant) and plant height (cm) at the end of the experiment following SWE Foliar (Fol) and Drench (Dre) treatments with 5 or 7 mL/plant. Values are expressed as mean of triplicate determinations ± sd

		Leaf number (leaf/plant)	Leaf area (cm2/plant)	Plant dry weight (g/plant)	Plant height (cm)
CHSD	CONTROL	34.6 ± 0.9b^a^	173 ± 2.1b	4.11 ± 0.1 b	21.5 ± 0.1 b
	(5 ml) Fol	35.1 ± 0.5b	178.6 ± 3.1b	4.18 ± 0.3 b	21.8 ± 0.1 a
	(7 ml)Fol	37.3 ± 0.3a	184.3 ± 4.2ab	4.23 ± 0.1 ab	21.9 ± 0.3a
	(5 ml)Dre	36.7 ± 0.7a	183.1 ± 3.5ab	4.31 ± 0.1 ab	22.4 ± 0.5 a
	(7 ml)Dre	37.9 ± 0.4a	193.5 ± 3.7a	4.42 ± 0.3 a	22.7 ± 0.3 a
CHSF	CONTROL	32.7 ± 0.5c	161 ± 1.7c	3.81 ± 0.2 b	19.7 ± 0.4 b
	(5 ml) Fol	33.3 ± 0.1bc	169 ± 3.2b	3.98 ± 0.1 b	20.3 ± 0.3 b
	(7 ml)Fol	34.9 ± 0.6bc	171.1 ± 2.5b	4.19 ± 0.1 ab	20.7 ± 0.2 b
	(5 ml)Dre	35.1 ± 0.2b	179 ± 1.7a	4.23 ± 0.2 a	21.1 ± 0.4 ab
	(7 ml)Dre	37.1 ± 0.3a	181.7 ± 3.3a	4.31 ± 0.1 a	21.9 ± 0.5 a

Notes: *CHSD C. h*. ʻ Superbells® Dreamsicleʼ, *CHSF C. h.* ʻSuperbells® Frost Fireʼ, *Dre* Drench treatment, *Fol* Foliar treatment

^a^Means followed by different letters within a column indicate significant differences between treatments based on LSD test (*P ≤ 0.05*)

**Table 2:** *Calibrachoa* cultivars leaves methanolic extracts DPPH and *β*-carotene-linoleic acid assay results as well as total phenolics, major flavonols and tannins contents following SWE Foliar (Fol) and Drench (Dre) treatments with 5 or 7 mL/plant. Values are expressed as mean of triplicate determinations ± sd

		DPPH free radical scavenging activity (IC50, ug/ml)	*β*-Carotenelinoleic acid assay (IC50, ug/ml)	Total phenolic content (mg GAE g-1 ext.)	Flavonols (mg g-1)		Tannins	
					quercetin-3-O-(caffeoyl diglucoside)	quercetin-3-O-(2-Ocaffeoyl6-Omalonyl diglucoside)	kaempferol 3-O-(feruloyl diglucoside)	(mg/ g-1 ext.)
CHSD	CONTROL	33 ± 0.5c^a^	31.1 ± 0.5c	4.6 ± 0.2 c	0.04 ± 0.01 c	ND^b^	0.30 ± 0.03 c	0.21 ± 0.004 c
	(5 ml) Fol	30.3 ± 0.7b	29.7 ± 0.3c	4.8 ± 0.2 c	0.09 ± 0.01 b	0.13 ± 0.01 b	0.42 ± 0.01 b	0.23 ± 0.001 c
	(7 ml)Fol	29.5 ± 0.6b	28.9 ± 0.5bc	4.9 ± 0.1 b	0.10 ± 0.01 b	0.12 ± 0.02 b	0.43 ± 0.03 b	0.25 ± 0.005 b
	(5 ml)Dre	28.5 ± 0.9ab	27.3 ± 0.5b	5 ± 0.3 b	0.14 ± 0.01 a	0.21 ± 0.02 a	0.52 ± 0.03 a	0.27 ± 0.01 ab
	(7 ml)Dre	27.3 ± 0.3a	25.5 ± 0.3a	5.2 ± 0.1 a	0.15 ± 0.02 a	0.22 ± 0.03 a	0.53 ± 0.01 a	0.29 ± 0.01 a
CHSF	CONTROL	37.9 ± 0.3c	34.3 ± 0.3c	4.4 ± 0.1 c	0.07 ± 0.01 d	0.11 ± 0.01 e	0.24 ± 0.01 d	0.10 ± 0.001 c
	(5 ml) Fol	34.9 ± 0.3bc	31 ± 0.1b	4.6 ± 0.08 bc	0.12 ± 0.02 c	0.14 ± 0.02 d	0.27 ± 0.01 c	0.12 ± 0.001 bc
	(7 ml)Fol	33.6 ± 0.5bc	30.5 ± 0.3b	4.8 ± 0.1 b	0.15 ± 0.01 b	0.16 ± 0.01 c	0.32 ± 0.02 b	0.14 ± 0.01 b
	(5 ml)Dre	32.2 ± 0.5b	28.9 ± 0.7ab	5 ± 0.1 a	0.22 ± 0.02 a	0.19 ± 0.01 b	0.34 ± 0.01 ab	0.16 ± 0.01 ab
	(7 ml)Dre	29.4 ± 0.1a	27.1 ± .5a	5 ± 0.2 a	0.23 ± 0.01 a	0.22 ± 0.01 a	0.35 ± 0.02 a	0.18 ± 0.01 a
	BHT	19.2 ± 0.1	17.3 ± 0.2					

Notes: *CHSD C. h*. ʻ Superbells® Dreamsicleʼ, *CHSF C. h.* ʻSuperbells® Frost Fireʼ, *Dre* Drench treatment, *Fol* Foliar treatment. Flavonols were expressed as mg/ g-1 ext

^a^Means followed by different letters within a column indicate significant differences between treatments based on LSD test (*P ≤ 0.05*)

^b^ND: not detected

Compared to untreated plants, SWE treatments significantly enhanced the flavonoid content of the leaves of both cultivars (Table 2). Increasing foliar SWE doses from 5 to 7 ml did not enhance the flavonoid content of leaf extracts in the CHSD cultivar. In CHSF, there were significant increases in the flavonoid contents with increasing SWE rates from 5 to 7 ml. In both cultivars, the drench application of SWE significantly enhanced the flavonoid content compared to the foliar application. Generally, increases were observed in tannin content of plants treated with SWE with different doses. Increasing SWE significantly enhanced the tannin content of the plants. Drench applications also resulted in higher tannin content. The mineral chemical analyses of SWE showed the presence of moderate percentage of nitrogen (N, 0.5 %) and phosphorus (P_2_O_5_, 0.19 %) and relatively high ratio of potassium (K_2_O, 0.7 %). Other important minerals were found such as magnesium (0.1 %), calcium (0.9 %). The heavy metals including iron, copper, zinc, manganese and boron were in trace ratios (1–5 × 10^−4^).

### Antifungal activities of leaf extracts

The two *Calibrachoa* cultivars showed significant antifungal activities when treated with SWEs compared to commercial fungicides (Table 3). Moreover, the application of SWE significantly enhanced leaf extract antifungal activities against different fungi. In CHSD, the control treatment showed higher antifungal activity than one of the fungicides used in this study. The MIC and MFC of the methanolic extracts were in the range of 0.07–0.29 mg/ml and from 0.16 to 0.42 mg/ml, respectively. SWE treatments significantly reduced the MIC values in leaf extracts against *A. flavus* from 0.16 mg/ml in the control to 0.08 mg/ml in the 7 ml drench treatment. Similar trends were found in *C. albicans*, *P. funiculosum* and *P. ochrochloron*. In addition, the MFC values were significantly reduced following SWE treatments and the MFC values following drench 7 ml treatments were the significantly lowest in all fungi. The foliar SWE treatments did not show significant differences against *C. albicans*, *P. funiculosum* and *P. ochrochloron*. The antifungal activities of the leaf extracts of CHSD plants treated with drench 7 ml SWE were higher than commercial fungicides.

**Table 3:** Minimum inhibitory (MIC) and fungicidal concentration (MFC) of the methanolic extract (mg/mL) of *Calibrachoa* cultivars contents following SWE foliar (Fol) and Drench (Dre) treatments with 5 or 7 mL/plant. Values are expressed as mean of triplicate determinations ± sd

	SWE treatments	*Aspergillus flavus*	*Candida albicans*	*Penicillium funiculosum*	*Penicillium ochrochloron*
CHSD	MIC				
	FLZ	0.16 ± 0.03b	0.11 ± 0.06c	0.15 ± 0.04c	0.19 ± 0.08c
	KTZ	0.20 ± 0.02a	0.23 ± 0.04a	2.00 ± 0.03a	0.20 ± 0.02c
	CONTROL	0.16 ± 0.002b	0.14 ± 0.007b	0.21 ± 0.002b	0.29 ± 0.001a
	(5 ml) Fol	0.15 ± 0.006bc	0.14 ± 0.003bc	0.21 ± 0.005b	0.29 ± 0.003a
	(7 ml)Fol	0.13 ± 0.002c	0.12 ± 0.003c	0.19 ± 0.006bc	0.25 ± 0.003b
	(5 ml)Dre	0.10 ± 0.001 cd	0.1 ± 0.004 cd	0.16 ± 0.005c	0.21 ± 0.002c
	(7 ml)Dre	0.08 ± 0.004d	0.07 ± 0.001d	0.14 ± 0.001c	0.18 ± 0.001c
	MFC				
	FLZ	0.21 ± 0.06c	0.16 ± 0.07d	0.24 ± 0.02d	0.25 ± 0.01d
	KTZ	0.40 ± 0.05a	0.40 ± 0.01a	3.45 ± 0.04a	0.39 ± 0.02a
	CONTROL	0.26 ± 0.001b	0.30 ± 0.001b	0.36 ± 0.001b	0.42 ± 0.001a
	(5 ml) Fol	0.25 ± 0.001b	0.30 ± 0.001b	0.36 ± 0.001b	0.42 ± 0.001a
	(7 ml)Fol	0.22 ± 0.001c	0.27 ± 0.001b	0.33 ± 0.001b	0.40 ± 0.001a
	(5 ml)Dre	0.19 ± 0.001 cd	0.22 ± 0.001c	0.29 ± 0.001c	0.34 ± 0.001b
	(7 ml)Dre	0.16 ± 0.001d	0.16 ± 0.001d	0.24 ± 0.001d	0.29 ± 0.001c
CHSF	MIC				
	FLZ	0.16 ± 0.03c	0.11 ± 0.06d	0.15 ± 0.04f	0.19 ± 0.08c
	KTZ	0.20 ± 0.02b	0.23 ± 0.04a	2.00 ± 0.03a	0.20 ± 0.02c
	CONTROL	0.25 ± 0.001a	0.23 ± 0.001a	0.31 ± 0.001b	0.31 ± 0.001a
	(5 ml) Fol	0.21 ± 0.001b	0.20 ± 0.001b	0.24 ± 0.001c	0.27 ± 0.001b
	(7 ml)Fol	0.19 ± 0.001bc	0.19 ± 0.001b	0.21 ± 0.001d	0.24 ± 0.001b
	(5 ml)Dre	0.17 ± 0.001c	0.16 ± 0.001c	0.18 ± 0.001e	0.20 ± 0.001c
	(7 ml)Dre	0.13 ± 0.001d	0.13 ± 0.001d	0.14 ± 0.001f	0.16 ± 0.001d
	MFC				
	FLZ	0.21 ± 0.06d	0.16 ± 0.07e	0.24 ± 0.02	0.25 ± 0.01f
	KTZ	0.40 ± 0.05a	0.40 ± 0.01b	3.45 ± 0.04a	0.39 ± 0.02d
	CONTROL	0.35 ± 0.001b	0.45 ± 0.001a	0.50 ± 0.001b	0.56 ± 0.001a
	(5 ml) Fol	0.29 ± 0.001c	0.36 ± 0.001bc	0.43 ± 0.001c	0.50 ± 0.001b
	(7 ml)Fol	0.28 ± 0.001c	0.33 ± 0.001c	0.39 ± 0.001d	0.46 ± 0.001c
	(5 ml)Dre	0.24 ± 0.001d	0.29 ± 0.001 cd	0.36 ± 0.001d	0.38 ± 0.001d
	(7 ml)Dre	0.22 ± 0.001d	0.26 ± 0.001d	0.30 ± 0.001e	0.29 ± 0.001e

Notes: *CHSD C. h*. ʻ Superbells® Dreamsicleʼ, *CHSF C. h.* ʻSuperbells® Frost Fireʼ, *Dre* Drench treatment, *Fol* Foliar treatment

*Means followed by different letters within a column indicate significant differences between treatments based on LSD test (*P ≤ 0.05*). The fungicides fluconazole (FLZ) and ketoconazole (KLZ) were used as positive controls

In CHSF, following SWE treatments there were significant reductions in MIC and MBC values and the values ranged from 0.13 to 0.31 mg/ml and from 0.22 to 0.56 mg/ml, respectively. In *A. flavus*, there were reductions in the MIC and MBC values from 0.25 to 0.13 mg/ml and from 0.35 to 0.22 mg/ml, respectively when treating the plants with 7 ml SWE as drench application compared to control plants. A similar trend was found in all fungi examined. Interestingly, the foliar treatments of SWE had significant antifungal effects compared to control treatments, but drench treatments generally had higher antifungal activities.

### Antibacterial activities of leaf extracts

Leaf extracts showed moderate to high antibacterial activities in both cultivars following SWE treatments (Table 4). In CHSD, the MIC and MBC of the methanolic extracts ranged from 0.06 to 0.18 mg/ml and from 0.10 mg/ml to 0.32 mg/ml, respectively. In *B. cereus*, SWE treatments significantly reduced the MIC values with increasing the amount from 5 to 7 ml in the foliar and drench treatments. The lowest MIC values were 0.07 mg/ml and 0.065 mg/ml in the plants treated with drench SWE applications with 5 and 7 ml, respectively. The drench 5 and 7 ml treatments reduced the MIC values lower than antibiotics. In *S. aureus*, *M. flavus* and *E cloacae*, there were reductions in the MIC values when increasing SWE rates from 5 to 7 ml and when using the drench SWE treatments. The MIC values in *S. aureus*, *M. flavus* and *E cloacae* were similar or slightly higher than antibiotics. The MBC values followed similar trend for that found in the MIC regarding the significant reduction in in the MBC values in plants treated with SWE compared to the control and the reduction of the MBC values when using drench SWE treatments.

**Table 4:** Minimum inhibitory (MIC) and bactericidal concentration (MBC) of the methanolic extract (mg/mL) of Calibrachoa cultivars contents following SWE foliar (Fol) and Drench (Dre) treatments with 5 or 7 mL/plant. Values are expressed as mean of triplicate determinations ± sd

	SWE treatments	*Bacillus cereus*	*Staphylococcus aureus*	*Micrococcus flavus*	*Enterobacter cloacae*
CHSD	MIC				
	Streptomycin	0.08 ± 0.004b	0.20 ± 0.01a	0.11 ± 0.01c	0.06 ± 0.001e
	Ampicillin	0.08 ± 0.003b	0.11 ± 0.01e	0.10 ± 0.02 cd	0.15 ± 0.03ab
	CONTROL	0.10 ± 0.001a	0.18 ± 0.002b	0.18 ± 0.001a	0.17 ± 0.002a
	(5 ml) Fol	0.09 ± 0.003ab	0.17 ± 0.007bc	0.17 ± 0.002ab	0.16 ± 0.001a
	(7 ml)Fol	0.08 ± 0.002b	0.16 ± 0.005c	0.15 ± 0.005b	0.14 ± 0.001b
	(5 ml)Dre	0.07 ± 0.001bc	0.14 ± 0.005d	0.12 ± 0.004c	0.10 ± 0.004c
	(7 ml)Dre	0.065 ± 0.003c	0.1 ± 0.02e	0.08 ± 0.002d	0.08 ± 0.002d
	MBC				
	Streptomycin	0.11 ± 0.002de	0.40 ± 0.003a	0.21 ± 0.01c	0.10 ± 0.03d
	Ampicillin	0.19 ± 0.003a	0.17 ± 0.002 g	0.20 ± 0.01c	0.21 ± 0.002c
	CONTROL	0.17 ± 0.005b	0.30 ± 0.001b	0.30 ± 0.001a	0.32 ± 0.001a
	(5 ml) Fol	0.15 ± 0.001c	0.28 ± 0.003c	0.28 ± 0.004ab	0.31 ± 0.005a
	(7 ml)Fol	0.14 ± 0.004c	0.26 ± 0.004d	0.25 ± 0.001b	0.27 ± 0.004b
	(5 ml)Dre	0.12 ± 0.005d	0.23 ± 0.005e	0.22 ± 0.002c	0.22 ± 0.003c
	(7 ml)Dre	0.10 ± 0.002e	0.21 ± 0.001f	0.16 ± 0.005d	0.19 ± 0.001c
CHSF	MIC				
	Streptomycin	0.08 ± 0.004c	0.20 ± 0.01c	0.11 ± 0.01e	0.06 ± 0.001e
	Ampicillin	0.08 ± 0.003c	0.11 ± 0.01e	0.10 ± 0.02e	0.15 ± 0.003d
	CONTROL	0.14 ± 0.001a	0.26 ± 0.004a	0.25 ± 0.001a	0.28 ± 0.001a
	(5 ml) Fol	0.13 ± 0.002a	0.23 ± 0.005b	0.22 ± 0.002b	0.25 ± 0.005b
	(7 ml)Fol	0.11 ± 0.000b	0.19 ± 0.001c	0.19 ± 0.001c	0.22 ± 0.004c
	(5 ml)Dre	0.10 ± 0.002b	0.17 ± 0.001 cd	0.18 ± 0.005 cd	0.20 ± 0.002c
	(7 ml)Dre	0.08 ± 0.005c	0.15 ± 0.002d	0.15 ± 0.001d	0.16 ± 0.001d
	MBC				
	Streptomycin	0.11 ± 0.002f	0.40 ± 0.003b	0.21 ± 0.01d	0.10 ± 0.03e
	Ampicillin	0.19 ± 0.003d	0.17 ± 0.002e	0.20 ± 0.01d	0.21 ± 0.002d
	CONTROL	0.31 ± 0.001a	0.45 ± 0.002a	0.47 ± 0.002a	0.45 ± 0.001a
	(5 ml) Fol	0.27 ± 0.002b	0.40 ± 0.001b	0.44 ± 0.001a	0.43 ± 0.002a
	(7 ml)Fol	0.24 ± 0.001c	0.38 ± 0.001b	0.39 ± 0.006b	0.37 ± 0.005b
	(5 ml)Dre	0.19 ± 0.002d	0.32 ± 0.002c	0.33 ± 0.001c	0.34 ± 0.001b
	(7 ml)Dre	0.16 ± 0.003e	0.29 ± 0.001d	0.30 ± 0.003c	0.28 ± 0.002c

Notes: *CHSD C. h*. ʻ Superbells® Dreamsicleʼ, *CHSF C. h.* ʻSuperbells® Frost Fireʼ, *Dre* Drench treatment, *Fol* Foliar treatment

*Means followed by different letters within a column indicate significant differences between treatments based on LSD test (*P ≤ 0.05)*

In CHSF, there were reductions in the MIC and MBC values when using the leaf extracts of SWE-treated plants. In *B. cereus*, the reduction in the MIC value was not significant when using 5 ml foliar SWE compared to the control. However, significant reductions were found when increasing SWE rate from 5 to 7 ml and when using drench SWE treatments. In *S. aureus*, *M. flavus* and *E cloacae*, there were significant reductions when using SWE foliar or drench treatments compared to the control. The MIC values in the plants treated with SWE matched those found in the antibiotics or were slightly higher. The MBC values followed similar trend for that found in the MIC values in CHSF. SWE treatments significantly reduced the MBC values compared to the control treatment. In addition, values for SWE treatments were close to those from the antibiotics treatments used in the experiment.

## Discussion

The improved growth in *Calibrachoa* plants following SWE treatments concord previous results on other plants [8, 9, 11]. In both cultivars, increases found in phenolic and flavonols contents following SWE treatments were associated with concurrent increases in antioxidant activity. These results are in agreement with previous studies on vegetable crops [8, 10, 11]. The chemical analyses of SWE showed some important major and minor nutrients available in SWE for the treated plants that might enhance their growth and secondary metabolite compositions. Several reports indicated that organic fertilizers [39], NPK mineral fertilizers [40], higher supplementation of potassium [41] enhance overall plant growth, the phenolic and flavonoid composition as well as the antioxidant activities. The increased phenolic and flavonoid content associated with increased antioxidant activities following SWE treatment of *Sargassum johnstonii* Setchell & Gardner [8] and *Ascophyllum nodosum* [10, 11]. In this study, SWE significantly enhanced plant leaf total phenolic, flavonols and tannin content that consequently increased the antioxidant activity of leaf extracts. Further, increases in the phenolic content of the leaves of the cuttings of *Pelargonium* were observed following SWE treatment [42]. However, one report found that the phenolic and flavonoid content might not increase following SWE treatment [43]. It is well documented that phenolic compounds are the main secondary metabolites in plants that are considered as the major antioxidant resource in horticultural crops [44]. In addition, the flavonoids as polyphenolic compounds are secondary metabolites that exhibit strong antioxidant activities [45–47]. The increased antioxidant activities associated with increased tannins in leaf extracts is in agreement with previous reports that highlighted the role of tannins in enhancing the overall antioxidant values of plant extracts [48, 49]. The drench applications of SWE enhanced the secondary metabolite composition of both *Calibrachoa* cultivars compared to foliar application and control plants. This finding is in agreement with previous reports showing that foliar and drench applications of SWE may result in diverse effects on crops [7–9]. Positive differences were found in this study in almost all parameters among plants treated with different doses of SWE. Mattner et al. [50] found that soil drench application of SWE enhanced the vegetative growth of broccoli; also, it was found that SWE soil drench doses increased the leaf area in one orange cultivar but had no effect on dry weight and stem dry weight compared to foliar applications [7].

We found that the increase in the phenolic, flavonols and tannin content was associated with enhanced antifungal and antibacterial activities. The enhanced antifungal activities against *A. flavus*, *C albicans*, *P. funiculosum* and *P. ochrochloron* were observed following treatment with extracts of plants sprayed or soil drenched with SWE; this may be attributed to the increased contents of phenols [51, 52]. Yazdani et al. [53] reported that certain phenolic compounds isolated from the methanolic extracts of *Piper betle* L. (Piperaceae) could inhibit the growth of *A. flavus*. Phenolics isolated from the root bark of *Lycium chinense* Miller (Solanaceae) [54], the leaves of *Baseonema acuminatum* (Apocynaceae) [55], and the leaves of *Hyssopus officinalis* [56] have been associated with the antifungal and antioxidant activities. The bioactivity of phenolic compounds might be attributed to the interferece with proteins and forming non-covalent bonds leading to conformation changes and protein inactivation in microbes [57]. Hussin et al. [58] reported that *Barringtonia racemosa* L. (Lecythidaceae) leaf extracts have strong antifungal activities against *Aspergillus* sp. and *Penicillium* sp. as well as other fungi which they explained by the presence of four different flavonoids and two phenolic acids. In the current study, increased specific flavonols associated with increased antifungal activities which may agree with previous reports that specific phenolic compounds were responsible for antifungal activity [59]. Some reports even indicate that tannins might be responsible for the antifungal activity of plant extract such as that reported from the fruit peels of *Punica granatum* which mainly contains tannins and was efficient against *Aspergillus niger* and *Penicillium citrinum* [60]. The tannins in the current study significantly increased in SWE treated plants, which support previous investigations.

Observed antibacterial activities following SWE treatments might be attributed to increases in phenolic compounds which are commonly reported in antibacterial plant extracts [61, 62]. A wide range of studies have provided support for this. For example, Stanković et al. [63] found that phenolic and flavonoids compounds found in leaf extracts of *Teucrium* sp. have strong antibacterial activities against *Staphyllococcus aureus*, *Pseudomonas aeroginosa* and *E. coli*, with *S. aureus* being the most sensitive. Similarly, Nitiema et al. [64] found that specific phenolics such as coumarins have antibacterial activities against a wide spectrum of organisms such as *Enterobacter aerogens*. Also Vaquero et al. [65] reported that the antimicrobial property of different wines depends on the presence of pure phenolic compounds and polyphenols, and that clarified wines were inactive against all bacteria. What’s more, Edziri et al. [59] found that two flavonoids were responsible for antibacterial activity against *Pseudomonas aeruginosa* and *Escherichia coli* (7.81–15.62 μg/ml). Furtheremore, Dahham et al. [60] reported that pomegranate fruit peelings, which mainly contain tannins, have strong antibacterial activity against *S. aureus* and moderate antibacterial activity against *Bacillus cereus*. Finally, Saravanakumar et al. [66] reported that *Thespesia populnea* flower extracts showed strong antibacterial activity against wide spectrum of species including *Micrococcus flavus* due to the presence of flavonoids and phenols in the extracts.

In the present study, SWE as biostimulant that contain important nutrient composition might boost the vegetative growth and secondary metabolite composition of *Calibrachoa* plants that might enhance their respective bioactivity against microorganisms. Diverse responses were found among fungi and bacteria. The most sensitive fungus was *C. albicans* and the most sensitive bacterium was *E. cloacae* while the most resistant fungus was *P. ochrochloron* and the most resistant bacterium was *S. aureus*. CHSD showed higher antioxidant activities than CHSF due to higher phenols, flavonols and tannins content. The antioxidant activities found in this study matches those found in *Petunia* [67, 68] and also are in agreement with recent studies as response to SWE [69, 70]. The cultivars CHSD and CHSF showed enhanced antibacterial and antifungal activities following SWE treatments, and this implies that SWE treatments might be used to enhance the medicinal values of these plants and their use as potential alternatives for antibiotics and commercial reagents to protect human health and the environment.

## Conclusion

*Ascophyllum nodosum* SWE treatments significantly enhanced plant’s vegetative growth as well as production of bioactive molecules such as the phenolics and flavonoids, which ultimately enhanced the antifungal and antibacterial activities of the leaf extracts. Hence, the application of SWE on *Calibrachoa* cultivars might be useful in increasing the medicinal value of these plants and help produce a natural alternative to antibiotics and fungicides. Finally, the application of SWE as a drench at 7 ml/L showed significant differences in the improvement of chemical composition and bioactivity when compared to the foliar application, suggesting that the method of application can alter the composition of natural products within plants.
